# Genetic Characterisation of Malawian Pneumococci Prior to the Roll-Out of the PCV13 Vaccine Using a High-Throughput Whole Genome Sequencing Approach

**DOI:** 10.1371/journal.pone.0044250

**Published:** 2012-09-10

**Authors:** Dean B. Everett, Jennifer Cornick, Brigitte Denis, Claire Chewapreecha, Nicholas Croucher, Simon Harris, Julian Parkhill, Stephen Gordon, Enitan D. Carrol, Neil French, Robert S. Heyderman, Stephen D. Bentley

**Affiliations:** 1 Malawi-Liverpool-Wellcome Clinical Research Programme, Chichiri, Blantyre 3, Malawi; 2 Institute of Infection and Global Health, University of Liverpool, Liverpool, United Kingdom; 3 The Wellcome Trust Sanger Institute, Wellcome Trust Genome Campus, Cambridge, United Kingdom; 4 The Liverpool School of Tropical Medicine, Liverpool, United Kingdom; 5 Department of Women’s and Children’s Health, Institute of Child Health, University of Liverpool, Liverpool, United Kingdom; Baylor College of Medicine, United States of America

## Abstract

**Background:**

Malawi commenced the introduction of the 13-valent pneumococcal conjugate vaccine (PCV13) into the routine infant immunisation schedule in November 2011. Here we have tested the utility of high throughput whole genome sequencing to provide a high-resolution view of pre-vaccine pneumococcal epidemiology and population evolutionary trends to predict potential future change in population structure post introduction.

**Methods:**

One hundred and twenty seven (127) archived pneumococcal isolates from randomly selected adults and children presenting to the Queen Elizabeth Central Hospital, Blantyre, Malawi underwent whole genome sequencing.

**Results:**

The pneumococcal population was dominated by serotype 1 (20.5% of invasive isolates) prior to vaccine introduction. PCV13 is likely to protect against 62.9% of all circulating invasive pneumococci (78.3% in under-5-year-olds). Several Pneumococcal Molecular Epidemiology Network (PMEN) clones are now in circulation in Malawi which were previously undetected but the pandemic multidrug resistant PMEN1 lineage was not identified. Genome analysis identified a number of novel sequence types and serotype switching.

**Conclusions:**

High throughput genome sequencing is now feasible and has the capacity to simultaneously elucidate serotype, sequence type and as well as detailed genetic information. It enables population level characterization, providing a detailed picture of population structure and genome evolution relevant to disease control. Post-vaccine introduction surveillance supported by genome sequencing is essential to providing a comprehensive picture of the impact of PCV13 on pneumococcal population structure and informing future public health interventions.

## Introduction

Approximately 1.1 million deaths annually are attributed to invasive pneumococcal disease (IPD) worldwide, accounting for 9% of all deaths in developing countries [1] In Malawi the pneumococcus is a leading cause of pneumonia, bacteraemia and meningitis in both children and adults, and consequently a prominent cause of death [2], [3], [4]. Vaccine prevention of pneumococcal disease is therefore a public health priority.

Routine pneumococcal vaccination with PCV13 was introduced as part of the infant expanded programme of immunization in November 2011. Pneumococcal vaccine was not licensed or routinely available before this introduction. The vaccine programme is expected to be highly successful but based on experience in Europe and the United States, rapid epidemiological change is likely to follow vaccine introduction [5]. We have previously described 10 years of clinical phenotypic surveillance of pneumococcal invasive disease, which detailed a persistent decline in admissions with IPD to a large district and tertiary referral hospital in Blantyre, Malawi [6]. We have highlighted that pneumococcal epidemiology in Malawi is undergoing change prior to the introduction of vaccine.

Understanding this change in epidemiology requires reproducible and robust molecular typing methods. A range of techniques have been used, these include serotyping, antimicrobial resistance phenotyping and multilocus sequence typing (MLST) [7], [8], [9], [10], [11], [12], [13]. However, because of the highly diverse and recombinogenic nature of the pneumococcus, these approaches may not provide sufficient resolution and adequately predict change over time in complex populations. Whole genome sequencing is rapidly becoming an economically viable option for genetic analysis of large collections of bacterial isolates representing natural populations [14], [15], [16] giving unprecedented resolution on the individual genetics and relationships between strains. To provide a preliminary picture of invasive pneumococcal populations in Malawi and inform post-introduction surveillance of pneumococcal disease, we have therefore characterised a sample of 127 invasive pneumococcal isolates from 2003–2008 using high throughput whole genome sequencing.

## Results

**Figure 1:**
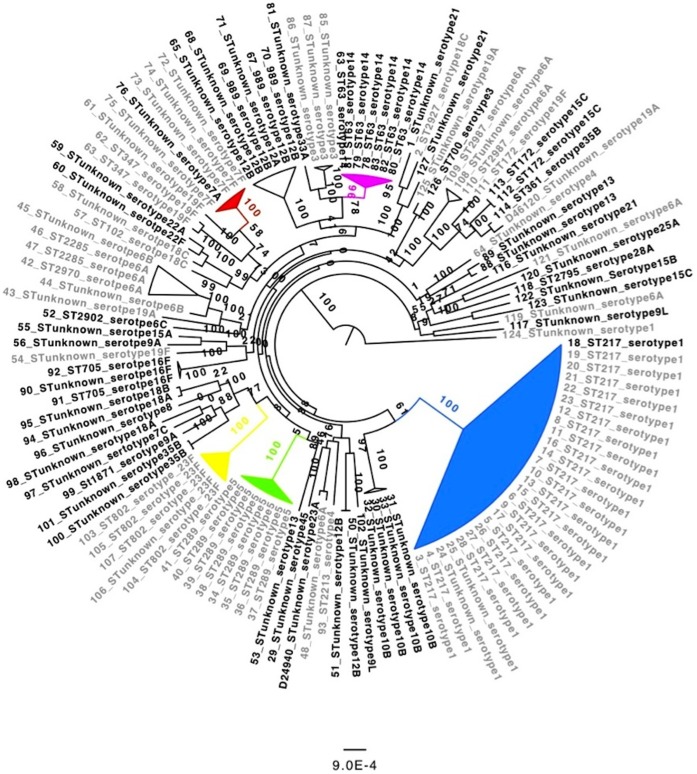
Phlyogenetic tree of Malawian strains.

Whole genome assemblies were generated for all 127 isolates and the protein sequences of 388 shared orthologous genes extracted and aligned to create the phylogenetic tree represented in Figure 1. Within the tree there are several clusters of similar sequence, which can be correlated with other characterizing metrics such as serotype and drug resistance profile. It is also notable that even for very closely related isolates within clusters it is possible, using the whole genome data, to specifically distinguish every isolate. Multi-locus sequence type (MLST) was derived from the sequence data to allow comparison of the genetic diversity of this sample with other samples in the literature and the entire public MLST database.

### Serotype Distribution and Pneumococcal Conjugate Vaccine Coverage

The serotype of each isolate was determined by mapping Illumina reads against a reference of concatenated capsular biosynthesis loci (*cps*) for all known serotypes (Figure 1). There were thirty-nine (39) circulating serotypes amongst the isolates: 22 were found in invasive isolates only, 1 from carriage only (15A) and 14 from both invasive disease and carriage. Six isolates (3, 5, 7F, 18C, 19A, 23F) were only associated with invasive disease and had population frequencies of greater than 3, but all are included in PCV13. Serotypes 13, 21 and 35B were more frequent in carriage than invasive disease. There were no non-typeable isolates amongst the carriage group. There were clusters though of serotypes 1, 3, 5, 6A/B, 7A/F, 10B, 12A/B, 14 and 23F correlating with genotypes previously reported [17].

**Figure 2:**
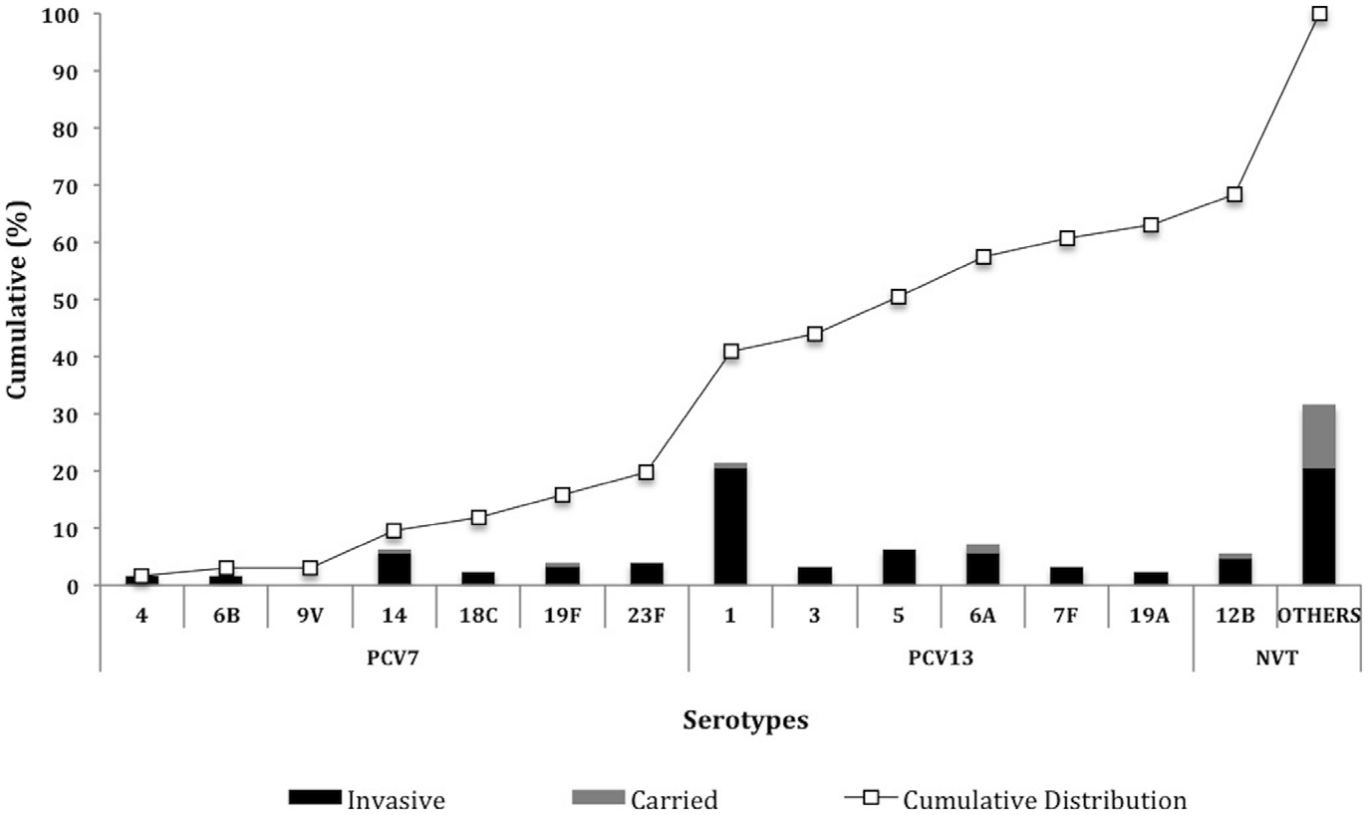
Bar chart showing rank order and cumulative serotype distribution (%).

Figure 2 details the cumulative distribution of serotypes detected directly from the genome in this study. The most common invasive serotypes in our selection were serotypes 1 (20.5%), 5 (6.3%), 6A (5.5%), 14 (5.5%), 12B (4.7%), 23F (3.9%) and 19F (3.1%), accounting for 49.5% of the serotypes detected within the population. Our data suggests that the 13-valent pneumococcal conjugate vaccine (PCV13) would provide 62.9% (95% CI 54.5 - 71.3%) coverage against all IPD in Malawi. However the vaccine is scheduled for children under-5 years old. 43.4% (46/106) of invasive isolates were from this age group and PCV13 coverage would be 78.3% (36/46).

### Antibiotic Resistance

If we remove the PCV13 serotypes from the analysis, 22.9% (8/35) of non-vaccine serotypes would be classified as multidrug resistant (MDR) by being resistant to at least 3 classes of antibiotics (File S1) and 25.7% (9/35) would be resistant to at least 2 classes of antibiotic. Drug resistance is clustered within the 10B, 12B and 16F carried and invasive groups on the tree (Figure 1).

### Sequence Type

The population diversity as judged by ST comprised of 20 distinct clonal groups. Novel STs accounted for 29.9% (38/127) of the population and included novel STs derived for serotypes 6B, 7F, 13, 18A and 19A (Table 1).

**Table 1:** Description of Circulating Clones.

ST	PMEN	Serotypes																		Invasive	Carriage	Total	Adult	Child
		PCV13																						
		1	3	4	5	6A	6B	7F	9V*	14	18C	19A	19F**	23F	15C**	12B	7A***	7C***	Others					
ST217	27	24																		24	0	24	9	15
ST700			1																	1	0	1	0	1
ST2213				1																1	0	1	0	1
ST289	19				8															8	0	8	3	5
ST2790						1														0	1	1	1	0
ST2285						2														2	0	2	0	2
ST2987						2														1	1	2	2	0
ST63	25									6										5	1	6	1	5
ST2678										1										1	0	1	0	1
ST2927											1									1	0	1	1	0
ST102											1								1	1	0	1	0	1
ST347													2							2	0	2	0	2
ST172													1		2					1	2	3	2	1
ST802														3						3	0	3	0	3
ST989																3				3	0	3	1	2
ST2902																			1	1	0	1	0	1
ST705																			1	1	1	2	1	1
ST1871																			1	1	0	1	0	1
ST361																			1	1	0	1	0	1
ST2795																			1	0	1	1	1	0
Unknown			3			3	2	4				2	1			1	1	1	19	31	7	38	18	20
Incomplete		3		1		1						1	1	2	1	1			10	17	7	24	12	12
																				**106**	**21**	**127**	**52**	**75**

* Not detected.

** Serotype switch.

*** Serotype switch.

The Pneumococcal Molecular Epidemiology Network (PMEN) 27 clone (Sequence Type [ST] 217) was exclusively associated with serotype 1 and PMEN 19 (ST289) with serotype 5 (Table 1). The 23F cluster corresponded to ST802; for two isolates within that cluster we could not assemble complete MLST genes but the whole genome data allows us to confidently place them within the ST802 group. Similarly one 12B sits within the ST989 group and a single 16F sits within the ST705 group (Figure 1). Two novel ST, serotype 12B isolates, cluster together, but not with the larger ST989 grouping. The serotype 14 cluster was predominately ST63 (75%), with a single ST2678 and a single novel ST. ST63 represents the PMEN 25 clone, which is known to express serotype 15A. However only serotypes 15B and 15C were identified of the serotype 15 cluster. The cluster of serotype 10B isolates did not correspond to a known MLST, but three of this cluster were from invasive serotypes and accounted for 2.4% of the population. No members of the pandemic 23F clone first identified in Spain (PMEN1, 23F^-Spain^, ST81) were detected [18], [19], [20].

### Lineage-serotype Associations

**Figure 3:**
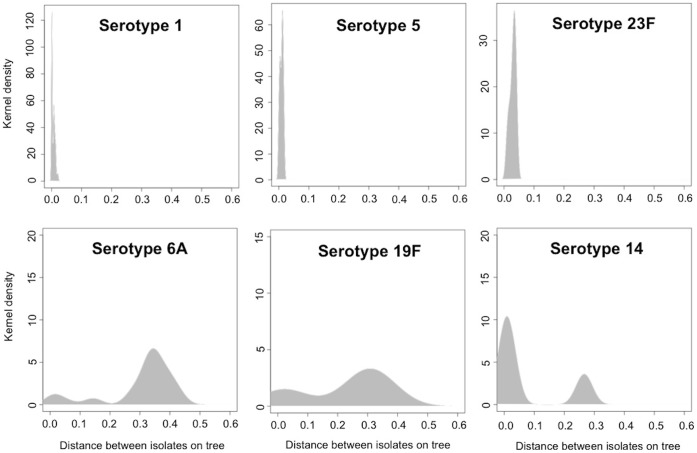
Genomic diversity of serotypes targeted by PCV13.

Figure 3 provides an overall summary of the genomic diversity associated with the serotypes targeted by the PCV13; Serotypes 1, 5 and 23F were exclusively detected from single lineages (distance of isolates falling within 0–0.002 amino acid changes per site), while some serotypes were detected from multiple lineages (distance >0.002 amino acid substitution per site). The vaccine will target more than one lineage associated with 3 serotypes (6A, 14 and 19F), which will potentially have different propensities to cause disease or association with antibiotic resistance. Lineage-serotype associations allow for the potential detection of serotype switch events; within this sample we found two isolates with the same ST (ST172) but unrelated serotypes (19F and 15C) indicating a likely serotype change within this lineage due to exchange of the *cps* locus via recombination. However, the genomic distance indicated by the branch lengths shows that these isolates are not as closely related as may be assumed by the common MLST type suggesting that the serotype switch is not a recent event and that there are likely to be many further genetic differences between these isolates. The serogroup 7 cluster appears to indicate a more recent switch event from serotype 7F to 7A. The difference between these serotypes is subtle, due to a single base insertion/deletion causing a frameshift in a glycosyl transferase gene thus altering the sugar composition of the repeating unit (PMID: 17766424). Our current assemblies of this specific location are not conclusive so further confirmation is required, nevertheless, the subtlety of the variation suggests that 7A/7F switches will be relatively frequent in nature.

### Carriage

There were 17 serotypes identified within this group and only 3 serotypes (6A, 7F and 19F) from 4 isolates (4/21, 19.1%) were included in the PCV13. Seven STs were identified, 7 were designated novel and 7 were incomplete and unable to determine. A single PMEN 25 (ST63) clone expressing serotype 14 was identified in this group. Carriage isolates were evenly distributed throughout the tree with no apparent clustering.

## Discussion

Until recently serotyping, antimicrobial resistance phenotyping and MLST have formed the basis for describing pneumococcal populations. Here we show that high throughput sequencing provides a comprehensive description of invasive pneumococci in Malawi through a single technique.

### Serotype Distribution

Deriving serotype from the genome data has allowed us to accurately define a serotype and serotype distribution of circulating strains in Malawi and predict the coverage of the conjugate vaccine (Figure 2). In total thirty-nine circulating serotypes were detected which is consistent with other African reports [21], [22]. Amongst the invasive pneumococcal isolates, serotype 1 predominated the circulating population with 20.5% of all invasive isolates attributable to this serotype. Serotype 5 (6.3%) was the next most commonly encountered invasive serotypes in our population. The dominance of serotype 1 and 5 has not changed in our setting over time, although the distribution across the population has reduced [23]. With the exception of 12B all the dominant serotypes observed in this study are included in the in PCV13. As the impact of the vaccine becomes more apparent and widespread it will therefore be essential to monitor the prevalence of 12B amongst invasive pneumococcal isolates. Across Africa, serotypes 1, 5 and 14 have also been commonly reported as major invasive serotypes [24]. In Europe the most common invasive serotypes have been reported as 6B, 14, 19F and 23F (quite different from the common Malawian serotypes) [25], however taking these data together it appears that the breadth of the PCV13 is currently sufficient to substantially prevent IPD (outside of the original target populations of North America and Europe) [24], [25].

Nonetheless our data suggests that PCV13 would prevent 62.9% of all IPD cases and 78.3% of cases in under 5 year olds in Malawi. This was not unexpected because coverage concerns in our setting have been previously reported [23]. This compares with almost 90% prior to the vaccine roll out in The United States [26], [27]. The coverage amongst Malawian isolates is conditional on a strong protective effect against serotype 1. Previous formulations have provided uncertain protection against this important serotype [28]. Without serotype 1 efficacy PCV13 coverage in Malawi could be as low as 42.4%. These differences highlight the need for broadening the vaccines coverage to include serotypes in circulation within SSA either by increasing the valency by the development of protein based vaccines or through a combination of the two [29]. The results of this study have led us to undertake a much broader prospective carriage study to collect more data on a much larger collection of isolates as the impact of vaccination increases in the coming years.

### Antibiotic Resistance and Vaccine Serotypes

22.9% (8/35) of non-vaccine serotypes were multidrug resistant (MDR) and 25.7% (9/35) were resistant to at least 2 classes of antibiotic (as determined by conventional disc testing - File S1). All serotype 12B isolates were resistant to at least 2 classes of antibiotics, 50% of these were MDR. The introduction of PCV13 therefore may drive the emergence of antibiotic resistant 12B and other MDR non-vaccine serotypes, such as 16F and warrants monitoring over the coming years.

### Sequence Type

By deriving the STs genomically we were able to assign 89/127 (70.1%) isolates to 20 known STs (Table 1). Of note, 38/127 (30%) isolates were assigned to novel STs. By analysing whole genome phylogeny, we are able to confidently predict that 15.8% (6/38) of our novel STs will be situated within known clonal groups. Based on these predictions, we estimate that 84.2% (32/38) of the novel STs defined by the MLST website are truly novel. This clearly demonstrates the huge genetic diversity amongst the circulating Malawian invasive pneumococcal population. Of the known STs, unsurprisingly ST217 (all serotype 1) is the predominant ST in circulation, accounting for 18.9% (24/127) of the total.

We identified three clones amongst the Malawian isolates previously identified as PMEN clones 19 (ST289), 25 (ST63) and 27 (ST217), as having a wide geographic distribution (Table 1 and www.sph.emory.edu/PMEN). These clones appear established in Malawi given their frequency across the entire sampling period (2003–2008, File S1). No members of the pandemic Spanish 23F clone (PMEN1, 23F^-Spain^, ST81) were detected.

MLST is an excellent tool, but can be heavily biased because of the tendency to focus on the dominant lineages in typing studies and therefore these are the lineages that are submitted [17]. Routine whole genome sequencing has the potential to be less biased as evidenced by the increased proportion of novel STs identified in this population and the ability to place unknown STs within particular serotype and ST groupings. In addition, analysis of the MLST genes can be used to determine ST thus allowing comparison with previously collected genotypic data described in the MLST database and in the literature. Analysis of the MLST database (www.mlst.net) showed similarities across the STs of the predominant Malawian serotypes and West Africa, but not to its nearest neighbours (Mozambique, Kenya or South Africa). This emphasizes the need to routinely deposit all isolates from routine surveillance in order to maximize the utility of this resource.

### Genotype

Genotypic analysis revealed a large degree of genetic heterogeneity within our population. To highlight future application of the vaccine in 2011, serotypes targeted by PCV13 were shaded in grey in Figure 1. Here, a serotype switch and a replacement of non-vaccine type may be predictable. An example highlighted in red is a cluster of unknown lineage associated with a carriage, a pneumonia and bacteraemic cases. Vaccine implementation would remove all 7F but leave bacteraemia-associated 7A of the same lineage. A similar scenario could occur with other serotypes. These techniques provide a unique opportunity to understand the pressures vaccine introduction will place on the pneumococcus and provide insights, which will inform this and future interventions.

Figure 3 illustrates that some serotypes were exclusively detected from single lineages, while some serotypes were detected from multiple lineages. Serotypes distributed within single lineages include 1 and 5 (left and middle diagrams in middle panel), which are exclusively associated with ST217 and ST289 respectively. Serotype 23F, slightly right shifted, were carried by ST802 and new STs. However, serotypes 14, 6A and 19F were detected in more than one lineage as shown by a right shift towards higher diversity in phylogenetic tree (bottom panel).

Following the introduction of PCV7 pneumococcal population structure within many countries changed by removing previously dominant clones. Methods of escape ensued resulting in serotype switching for example. In our current invasive population by employing the genome sequence data to derive phylogenetic relationships predict serotype and visualize lineage-serotype associations, we identified isolates with the same ST (ST172) but unrelated serotypes (19F and 15C). Thus indicating a likely serotype change within this lineage due to exchange of the *cps* locus via a recombination and also demonstrated the potential to detect more subtle serotype switches. Based on the clustering on the phylogenetic tree (Figure 1) predict that following full and stable coverage of PCV13, isolates expressing serotype 7A could switch to 7F, 19Fs could switch to 15C and serotypes 10B, 12B and 16F could emerge as major invasive drug resistant serotypes.

### Summary

Pneumococcal infections place a high social, health and economic burden on the infrastructure of resource-poor countries [30]. Whole genome sequencing provides a precise measure of population change over time, which was previously difficult and time consuming to achieve. It provides a greater population resolution than individually derived data extraction methods and a means to not only describe the pneumococcal population with unprecedented genetic detail, but it also provided the means to accurately detect and predict important changes within circulating clonal complexes, serotype distribution and serotype switches as the vaccine is implemented.

## Methods

### Ethics

The anonymised isolates characterised in this study are all clinical isolates from specimens obtained from Malawian adults and children as part of routine clinical diagnosis and management when they were admitted to QECH. The main ethical issue relates to specific consent for detailed characterisation of an isolate from a clinical specimen taken from a patient on clinical grounds. This requirement was waived by the University of Malawi, College of Medicine Research & Ethics Committee because the characterisation formed part of routine clinical management. The data are therefore published with the approval of the Research & Ethics Committee and conform to institutional guidelines.

### Setting

Queen Elizabeth Central Hospital (QECH) is a 1250 bed government-funded hospital, serving a population of ∼1 million, including the city of Blantyre, the surrounding townships, and outlying villages.

### Pneumococcal Isolate Selection

Isolation methodology and sensitivity testing protocols has been previously described [6]. Antimicrobial resistance but not serotyping was routinely performed on all isolates. The Malawi-Liverpool-Wellcome Trust Clinical Research Programme (MLW) has archived over 5000 carried and invasive pneumococcal isolates since 1996. One hundred and thirty-four invasive and carried isolates (134, 4.2%) were randomly selected (age range 1 day to 63 yrs) within individual years between 2003–2008 from a total of 3179 available isolates for this study. The rationale for the number is derived from the number of sequencing lanes we were offered by WTSI for the sequencing pilot (19 lanes, each lane had a capacity for 7 isolates = 133). An additional isolate as sent in error, which was subsequently sequenced and included.

However data is only available for 127 strains (106 invasive and 21 carriage) due to either insufficient quality of DNA or final sequence reads. The selection comprised of adult and paediatric bacteraemic and meningitis isolates. The number of carried isolates corresponded to 10% of those available within the time frame. The remainder (134-21 = 113) were allocated to the invasive group. In total 4.6% of all isolates available in that specified time frame were sequenced.

### Bacterial Culture and Genomic DNA Preparation

Liquid medium (Todd-Hewitt broth) was inoculated and grown overnight at 37°C. Genomic DNA preparation was performed using the Promega Wizard Genomic DNA Purification Kit (Promega, USA, Product Number A1125) A minimum of 50 µl of extracted DNA with a concentration 20 ng/µl and in a volume of 100 µl, and in fragments >10 kb was shipped for sequencing.

### Whole Genome Sequencing and Sequence Analysis

Sequencing: Index tagged libraries for each *S. pneumoniae* isolate were prepared, pooled and sequenced on the Illumina Genome Analyzer GAII as described previously [14]. Short reads were assembled using Velvet v1.0.03 [31], and ordered relative to a complete reference genome sequence (Accession number: FM211187) [32] using Abacus v2.5.1 [33].

Read mapping: Illumina sequence data as paired end reads with an insert size between 50 and 400 bp were mapped onto the reference genome *S. pneumoniae* ATCC 700669 (Accession number: FM211187) using SSAHA v2.2.1 [34], giving on average, 23x depth of coverage and more than 79.4% coverage of the reference genome.

Serotype and Sequence type: Serotype and MLST type were derived as described previously by Croucher *et al* [14].

### Phylogenetic Analysis

A phylogenetic tree of 127 pneumococcal genomes was created using RAxML v.7.2.8 [35] as described in Harris *et al* [15], [36], for all sites in the nucleotide sequences containing SNPs, using GAMMA correction and five starting trees. One-hundred (100) bootstraps were generated to validate the tree, which represents the best overall estimate of evolutionary history from our data.

Pair-wise distance between samples within a tree was used as a tool to investigate diversity within vaccine serotypes. We used R version 2.11.1 [37] to plot population density based on sample pair-wise distance for each serotype.

## Supporting Information

File S1 (DOCX)

## References

[1] O’Brien KL, Wolfson LJ, Watt JP, Henkle E, Deloria-Knoll M, et al (2009) Burden of disease caused by Streptococcus pneumoniae in children younger than 5 years: global estimates. Lancet 374: 893–902. 19748398 10.1016/S0140-6736(09)61204-6

[2] Carrol ED, Guiver M, Nkhoma S, Mankhambo LA, Marsh J, et al (2007) High pneumococcal DNA loads are associated with mortality in Malawian children with invasive pneumococcal disease. Pediatr Infect Dis J 26: 416–422. 17468652 10.1097/01.inf.0000260253.22994.61 PMC2810843

[3] Gordon S, Walsh A, Chaponda M, Gordon M, Soko D, et al (2000) Bacterial Meningitis in Malawian Adults: Pneumococcal Disease is Common, Severe, and Seasonal. Clinical Infectious Diseases 31: 53–57. 10913396 10.1086/313910

[4] Molyneux EM, Walsh AL, Forsyth H, Tembo M, Mwenechanya J, et al (2002) Dexamethasone treatment in childhood bacterial meningitis in Malawi: a randomised controlled trial. Lancet 360: 211–218. 12133656 10.1016/s0140-6736(02)09458-8

[5] Hicks LA, Harrison LH, Flannery B, Hadler JL, Schaffner W, et al (2007) Incidence of pneumococcal disease due to non-pneumococcal conjugate vaccine (PCV7) serotypes in the United States during the era of widespread PCV7 vaccination, 1998–2004. J Infect Dis 196: 1346–1354. 17922399 10.1086/521626

[6] Everett DB, Mukaka M, Denis B, Gordon SB, Carrol ED, et al (2011) Ten Years of Surveillance for Invasive Streptococcus pneumoniae during the Era of Antiretroviral Scale-Up and Cotrimoxazole Prophylaxis in Malawi. PLoS One 6: e17765. 21423577 10.1371/journal.pone.0017765 PMC3058053

[7] Enright MC, Spratt BG (1998) A multilocus sequence typing scheme for Streptococcus pneumoniae: identification of clones associated with serious invasive disease. Microbiology 144 (Pt 11): 3049–3060. 10.1099/00221287-144-11-3049 9846740

[8] Heineman HS (1973) Quellung test for pneumonia. N Engl J Med 288: 1027. 10.1056/NEJM197305102881921 4144492

[9] Lalitha MK, Pai R, John TJ, Thomas K, Jesudason MV, et al (1996) Serotyping of Streptococcus pneumoniae by agglutination assays: a cost-effective technique for developing countries. Bull World Health Organ 74: 387–390. 8823960 PMC2486887

[10] Lund E (1960) Laboratory diagnosis of Pneumococcus infections. Bull World Health Organ 23: 5–13. 14418893 PMC2555298

[11] Appelbaum PC (1992) Antimicrobial resistance in Streptococcus pneumoniae: an overview. Clinical infectious diseases : an official publication of the Infectious Diseases Society of America 15: 77–83. 1617076 10.1093/clinids/15.1.77

[12] Neufield F (1902) Ueberdie Agglutination der Pneumokokken and uber die Theorieen der Agglutination. Z Hyg Infektkrankh 40: 54.

[13] Sorensen UB (1993) Typing of pneumococci by using 12 pooled antisera. J Clin Microbiol 31: 2097–2100. 8370735 10.1128/jcm.31.8.2097-2100.1993 PMC265703

[14] Croucher NJ, Harris SR, Fraser C, Quail MA, Burton J, et al (2011) Rapid pneumococcal evolution in response to clinical interventions. Science 331: 430–434. 21273480 10.1126/science.1198545 PMC3648787

[15] Harris SR, Feil EJ, Holden MT, Quail MA, Nickerson EK, et al (2010) Evolution of MRSA during hospital transmission and intercontinental spread. Science 327: 469–474. 20093474 10.1126/science.1182395 PMC2821690

[16] He M, Sebaihia M, Lawley TD, Stabler RA, Dawson LF, et al (2010) Evolutionary dynamics of Clostridium difficile over short and long time scales. Proc Natl Acad Sci U S A 107: 7527–7532. 20368420 10.1073/pnas.0914322107 PMC2867753

[17] Donati C, Hiller NL, Tettelin H, Muzzi A, Croucher NJ, et al (2010) Structure and dynamics of the pan-genome of Streptococcus pneumoniae and closely related species. Genome Biol 11: R107. 21034474 10.1186/gb-2010-11-10-r107 PMC3218663

[18] McGee L, McDougal L, Zhou J, Spratt BG, Tenover FC, et al (2001) Nomenclature of major antimicrobial-resistant clones of Streptococcus pneumoniae defined by the pneumococcal molecular epidemiology network. J Clin Microbiol 39: 2565–2571. 11427569 10.1128/JCM.39.7.2565-2571.2001 PMC88185

[19] Munoz R, Coffey TJ, Daniels M, Dowson CG, Laible G, et al (1991) Intercontinental spread of a multiresistant clone of serotype 23F Streptococcus pneumoniae. J Infect Dis 164: 302–306. 1856478 10.1093/infdis/164.2.302

[20] Shi ZY, Enright MC, Wilkinson P, Griffiths D, Spratt BG (1998) Identification of three major clones of multiply antibiotic-resistant Streptococcus pneumoniae in Taiwanese hospitals by multilocus sequence typing. J Clin Microbiol 36: 3514–3519. 9817864 10.1128/jcm.36.12.3514-3519.1998 PMC105231

[21] Cutts FT, Zaman SM, Enwere G, Jaffar S, Levine OS, et al (2005) Efficacy of nine-valent pneumococcal conjugate vaccine against pneumonia and invasive pneumococcal disease in The Gambia: randomised, double-blind, placebo-controlled trial. Lancet 365: 1139–1146. 15794968 10.1016/S0140-6736(05)71876-6

[22] Klugman KP, Madhi SA, Huebner RE, Kohberger R, Mbelle N, et al (2003) A trial of a 9-valent pneumococcal conjugate vaccine in children with and those without HIV infection. N Engl J Med 349: 1341–1348. 14523142 10.1056/NEJMoa035060

[23] Gordon SB, Kanyanda S, Walsh AL, Goddard K, Chaponda M, et al (2003) Poor potential coverage for 7-valent pneumococcal conjugate vaccine, Malawi. Emerg Infect Dis 9: 747–749. 12781021 10.3201/eid0906.030020 PMC3000157

[24] Johnson HL, Deloria-Knoll M, Levine OS, Stoszek SK, Freimanis Hance L, et al.. (2010) Systematic evaluation of serotypes causing invasive pneumococcal disease among children under five: the pneumococcal global serotype project. PLoS Med 7. 10.1371/journal.pmed.1000348 PMC2950132 20957191

[25] Isaacman DJ, McIntosh ED, Reinert RR (2010) Burden of invasive pneumococcal disease and serotype distribution among Streptococcus pneumoniae isolates in young children in Europe: impact of the 7-valent pneumococcal conjugate vaccine and considerations for future conjugate vaccines. International journal of infectious diseases : IJID : official publication of the International Society for Infectious Diseases 14: e197–209. 19700359 10.1016/j.ijid.2009.05.010

[26] McIntosh ED, Reinert RR (2011) Global prevailing and emerging pediatric pneumococcal serotypes. Expert Rev Vaccines 10: 109–129. 21162625 10.1586/erv.10.145

[27] Pai R, Gertz RE, Beall B (2008) Invasive pneumococcal disease in children 5 years after conjugate vaccine introduction - eight states, 1998–2005. Sequential multiplex PCR approach for determining capsular serotypes of Streptococcus pneumoniae isolates. MMWR Morb Mortal Wkly Rep 57: 144–148.18272956

[28] Klugman K, Cutts F, Adegbola RA, Black S, Madhi SA, et al.. (2008) Meta - analysis of the efficacy of conjugate vaccines against invasive pneumococcal disease. In: Siber G, Klugman K, editors. Pneumococcal Vaccines: the Impact of Conjugate Vaccines. Washington, DC: ASM. 317–328.

[29] Paton JC, Boslego JW (2008) Pneumococcal vaccines: the impact of conjugate vaccine. In: Siber GR, Klugman KP, Mäkelä PH, editors. Protein vaccines. Washington, DC: ASM Press.

[30] All-Party Parliamentary Group on Pneumococcal Disease Prevention in the Developing World (2008) Improving global health by preventing pneumococcal disease. British Parliament. 1–35 p.

[31] Zerbino DR, Birney E (2008) Velvet: Algorithms for de novo short read assembly using de Bruijn graphs. Genome Research 18: 821–829. 18349386 10.1101/gr.074492.107 PMC2336801

[32] Croucher NJ, Walker D, Romero P, Lennard N, Paterson GK, et al (2008) The role of conjugative elements in the evolution of the multi-drug resistant pandemic clone Streptococcus pneumoniae Spain23F ST81. J Bacteriol 191: 1480–1489. 19114491 10.1128/JB.01343-08 PMC2648205

[33] Assefa S, Keane TM, Otto TD, Newbold C, Berriman M (2009) ABACAS: algorithm-based automatic contiguation of assembled sequences. Bioinformatics 25: 1968–1969. 19497936 10.1093/bioinformatics/btp347 PMC2712343

[34] Ning Z, Cox AJ, Mullikin JC (2001) SSAHA: A Fast Search Method for Large DNA Databases. Genome Research 11: 1725–1729. 11591649 10.1101/gr.194201 PMC311141

[35] Stamatakis A (2006) RAxML-VI-HPC: maximum likelihood-based phylogenetic analyses with thousands of taxa and mixed models. Bioinformatics 22: 2688–2690. 16928733 10.1093/bioinformatics/btl446

[36] Edgar RC (2004) MUSCLE: multiple sequence alignment with high accuracy and high throughput. Nucleic acids research 32: 1792–1797. 15034147 10.1093/nar/gkh340 PMC390337

[37] R Development Core Team (2010) R: A language and environment for statistical computing. Vienna, Austria: R foundation for Statistical Computing.

