# Supplementary material for: Genetic Characterisation of Malawian Pneumococci Prior to the Roll-Out of the PCV13 Vaccine Using a High-Throughput Whole Genome Sequencing Approach
Source: PLoS One. 2012 Sep 10;7(9):e44250. doi: 10.1371/journal.pone.0044250 (PMC3438182; doi:10.1371/journal.pone.0044250)
Supplement: File S1 — (DOCX) [file pone.0044250.s001.docx]

**Supplementary data:** Isolate details including ID, derived MLST, derived serotype, carriage/disease, year, adult/child

| ID on tree | ST | Serotype | Disease | Date Collected | Adult/Child |
| --- | --- | --- | --- | --- | --- |
| 1 | unknown | 21 | Carriage | Aug-04 | Adult |
| 2 | 2927 | 18C | Bacteraemia | 0ct-03 | Adult |
| 3 | 217 | 1 | Bacteraemia | May-07 | Child |
| 4 | 217 | 1 | Bacteraemia | Sep-04 | Adult |
| 5 | 217 | 1 | Meningitis | Nov-07 | Child |
| 6 | 217 | 1 | Bacteraemia | Apr-05 | Adult |
| 7 | 217 | 1 | Bacteraemia | May-05 | Adult |
| 8 | 217 | 1 | Bacteraemia | May-04 | Child |
| 9 | 217 | 1 | Bacteraemia | Dec-04 | Child |
| 10 | 217 | 1 | Meningitis | Nov-05 | Child |
| 11 | 217 | 1 | Bacteraemia | May-06 | Adult |
| 12 | 217 | 1 | Pneumonia | Aug-05 | Child |
| 13 | 217 | 1 | Bacteraemia | Oct-05 | Adult |
| 14 | 217 | 1 | Bacteraemia | Oct-05 | Child |
| 15 | 217 | 1 | Bacteraemia | Apr-07 | Adult |
| 16 | 217 | 1 | Meningitis | Mar-08 | Child |
| 17 | 217 | 1 | Meningitis | Dec-05 | Child |
| 18 | 217 | 1 | Meningitis | Apr-06 | Child |
| 19 | 217 | 1 | Meningitis | Oct-05 | Child |
| 20 | 217 | 1 | Meningitis | Apr-07 | Child |
| 21 | 217 | 1 | Bacteraemia | Jun-06 | Child |
| 22 | 217 | 1 | Meningitis | Feb-08 | Adult |
| 23 | 217 | 1 | Bacteraemia | Sep-08 | Child |
| 24 | incomplete | 1 | Bacteraemia | Sep-04 | Child |
| 25 | incomplete | 1 | Meningitis | Sep-06 | Child |
| 26 | 217 | 1 | Bacteraemia | Aug-04 | Adult |
| 27 | 217 | 1 | Bacteraemia | Sep-06 | Adult |
| 28 | 217 | 1 | Meningitis | Apr-04 | Child |
| 29 | unknown | 45 | Bacteraemia | Nov-06 | Adult |
| 30 | unknown | 10B | Bacteraemia | May-05 | Child |
| 31 | unknown | 10B | Carriage | Jul-04 | Adult |
| 32 | unknown | 10B | Meningitis | Feb-05 | Child |
| 33 | unknown | 10B | Bacteraemia | Oct-06 | Adult |
| 34 | 289 | 5 | Bacteraemia | Mar-04 | Adult |
| 35 | 289 | 5 | Bacteraemia | Nov-06 | Adult |
| 36 | 289 | 5 | Bacteraemia | Oct-06 | Adult |
| 37 | 289 | 5 | Bacteraemia | Nov-06 | Child |
| 38 | 289 | 5 | Meningitis | Oct-06 | Child |
| 39 | 289 | 5 | Meningitis | Aug-07 | Child |
| 40 | 289 | 5 | Meningitis | Mar-07 | Child |
| 41 | 289 | 5 | Pneumonia | Oct-04 | Child |
| 42 | 2790 | 6A | Carriage | Nov-05 | Adult |
| 43 | incomplete | 19A | Bacteraemia | Jul-06 | Adult |
| 44 | unknown | 6B | Bacteraemia | Mar-08 | Child |
| 45 | unknown | 6B | Bacteraemia | Apr-05 | Adult |
| 46 | 2285 | 6A | Bacteraemia | Jan-06 | Child |
| 47 | 2285 | 6A | Meningitis | Jun-08 | Child |
| 48 | unknown | 6A | Meningitis | Jun-06 | Child |
| 49 | unknown | 23A | Bacteraemia | Mar-04 | Child |
| 50 | incomplete | 12B | Meningitis | Jun-07 | Adult |
| 51 | incomplete | 12B | Meningitis | Jun-08 | Adult |
| 52 | 2902 | 6C | Meningitis | Apr-06 | Child |
| 53 | unknown | 13 | Carriage | Jun-06 | Adult |
| 54 | incomplete | 19F | Bacteraemia | Jun-05 | Child |
| 55 | incomplete | 15A | Carriage | Jul-05 | Adult |
| 56 | unknown | 9A | Bacteraemia | Mar-05 | Child |
| 57 | 102 | 18C | Meningitis | Sep-04 | Child |
| 58 | incomplete | 18C | Meningitis | Dec-05 | Child |
| 59 | unknown | 22A | Bacteraemia | Apr-04 | Adult |
| 60 | incomplete | 22F | Bacteraemia | Feb-05 | Adult |
| 61 | unknown | 19F | Meningitis | Jan-08 | Child |
| 62 | 347 | 19F | Bacteraemia | Jun-08 | Child |
| 63 | 347 | 19F | Meningitis | Aug-07 | Child |
| 64 | incomplete | 4 | Bacteraemia | Dec-05 | Adult |
| 65 | incomplete | 12B | Bacteraemia | May-06 | Child |
| 66 | unknown | 14 | Meningitis | May-04 | Child |
| 67 | 989 | 12B | Meningitis | May-04 | Child |
| 68 | unknown | 12B | Carriage | Oct-06 | Adult |
| 69 | 989 | 12B | Meningitis | Mar-08 | Adult |
| 70 | 989 | 12B | Meningitis | Nov-06 | Child |
| 71 | incomplete | 12A | Meningitis | Feb-08 | Child |
| 72 | unknown | 7F | Bacteraemia | Jun-08 | Child |
| 73 | incomplete | 7F | Carriage | Aug-05 | Adult |
| 74 | unknown | 7F | Meningitis | Oct-04 | Child |
| 75 | unknown | 7F | Pneumonia | May-05 | Child |
| 76 | unknown | 7A | Bacteraemia | Jan-08 | Adult |
| 77 | 63 | 14 | Meningitis | Mar-04 | Child |
| 78 | 63 | 14 | Meningitis | Dec-07 | Child |
| 79 | 63 | 14 | Bacteraemia | May-04 | Child |
| 80 | 63 | 14 | Carriage | Nov-06 | Adult |
| 81 | 63 | 14 | Meningitis | Sep-05 | Child |
| 82 | 63 | 14 | Meningitis | Nov-07 | Child |
| 83 | 2678 | 14 | Bacteraemia | May-06 | Child |
| 84 | unknown | 33A | Bacteraemia | Mar-08 | Adult |
| 85 | unknown | 3 | Bacteraemia | Oct-05 | Adult |
| 86 | unknown | 3 | Bacteraemia | Jul-05 | Adult |
| 87 | unknown | 3 | Bacteraemia | Oct-06 | Adult |
| 88 | unknown | 13 | Bacteraemia | Jun-05 | Child |
| 89 | unknown | 13 | Carriage | Jul-05 | Adult |
| 90 | incomplete | 16F | Bacteraemia | Jan-07 | Child |
| 91 | 705 | 16F | Carriage | May-05 | Adult |
| 92 | 705 | 16F | Bacteraemia | Mar-06 | Child |
| 93 | 2213 | 4 | Bacteraemia | Jul-06 | Child |
| 94 | unknown | 18A | Bacteraemia | Jul-04 | Adult |
| 95 | unknown | 18B | Bacteraemia | Jun-05 | Adult |
| 96 | incomplete | 8 | Bacteraemia | Jun-08 | Child |
| 97 | unknown | 7C | Carriage | May-04 | Adult |
| 98 | unknown | 18A | Carriage | Feb-03 | Adult |
| 99 | 1871 | 9A | Meningitis | Sep-04 | Child |
| 100 | incomplete | 35B | Carriage | Apr-04 | Adult |
| 101 | incomplete | 35B | Carriage | Oct-04 | Adult |
| 102 | unknown | 9L | Bacteraemia | Feb-07 | Child |
| 103 | incomplete | 23F | Bacteraemia | May-08 | Child |
| 104 | 802 | 23F | Bacteraemia | Jun-07 | Child |
| 105 | 802 | 23F | Meningitis | Nov-06 | Child |
| 106 | incomplete | 23F | Bacteraemia | Sep-07 | Child |
| 107 | 802 | 23F | Bacteraemia | Jul-04 | Child |
| 108 | unknown | 6A | Bacteraemia | Mar-06 | Child |
| 109 | 2987 | 6A | Carriage | Sep-04 | Adult |
| 110 | 2987 | 6A | Bacteraemia | May-02 | Adult |
| 111 | 172 | 19F | Carriage | Aug-06 | Adult |
| 112 | 172 | 15C | Carriage | Jul-06 | Adult |
| 113 | 172 | 15C | Bacteraemia | Feb-07 | Child |
| 114 | 361 | 35B | Bacteraemia | Jun-08 | Child |
| 115 | unknown | 19A | Bacteraemia | Mar-08 | Child |
| 116 | incomplete | 21 | Carriage | Oct-06 | Adult |
| 117 | unknown | 9L | Meningitis | Jun-05 | Child |
| 118 | 2795 | 28A | Carriage | Jul-04 | Adult |
| 119 | unknown | 6A | Bacteraemia | Oct-04 | Child |
| 120 | incomplete | 25A | Carriage | Apr-03 | Adult |
| 121 | incomplete | 6A | Bacteraemia | Jun-08 | Child |
| 122 | unknown | 15B | Bacteraemia | May-06 | Child |
| 123 | incomplete | 15C | Meningitis | Jun-06 | Child |
| 124 | incomplete | 1 | Carriage | Nov-06 | Adult |
| 125 | unknown | 19A | Bacteraemia | Jan-04 | Child |
| 126 | 700 | 3 | Bacteraemia | Feb-06 | Child |
| 127 | unknown | 21 | Meningitis | Apr-06 | Child |

**Supplementary data:** Antibiotic sensitivity in invasive non-vaccine serotypes

| ID on tree | Serotype | Penicillin | Chloramphenicol | Cotrimoxazole | Gentimicin | Erythromycin | Tetcycline | Ceftriaxone |
| --- | --- | --- | --- | --- | --- | --- | --- | --- |
| 52 | 6C | S | S | R | R | S | R | S |
| 76 | 7A | S | S | R | ND | S | R | S |
| 96 | 8 | S | S | S | ND | ND | S | ND |
| 56 | 9A | S | S | R | ND | S | S | ND |
| 99 | 9A | ND | ND | ND | ND | ND | ND | ND |
| 102 | 9L | R | S | R | ND | S | S | S |
| 117 | 9L | S | R | S | ND | ND | S | ND |
| 30 | 10B | S | S | R | R | S | R | S |
| 32 | 10B | S | R | S | ND | ND | R | ND |
| 33 | 10B | S | S | R | ND | S | R | S |
| 71 | 12A | S | R | R | ND | S | R | S |
| 50 | 12B | S | S | R | ND | S | S | S |
| 51 | 12B | S | S | R | ND | S | S | S |
| 65 | 12B | S | R | R | ND | S | R | S |
| 67 | 12B | S | R | R | R | S | R | S |
| 69 | 12B | S | S | R | ND | S | S | S |
| 70 | 12B | S | R | S | ND | ND | R | ND |
| 88 | 13 | S | S | S | ND | S | S | S |
| 122 | 15B | S | R | R | R | S | R | S |
| 113 | 15C | S | R | S | ND | ND | S | ND |
| 123 | 15C | S | S | R | R | S | S | S |
| 90 | 16F | R | R | S | ND | ND | R | ND |
| 92 | 16F | R | S | R | ND | S | R | S |
| 94 | 18A | S | S | R | ND | S | S | S |
| 95 | 18B | S | S | R | ND | S | S | S |
| 2 | 18C | R | S | R | ND | S | R | S |
| 57 | 18C | S | S | R | ND | S | R | S |
| 58 | 18C | S | R | S | ND | ND | S | ND |
| 127 | 21 | S | R | S | ND | ND | R | ND |
| 59 | 22A | S | S | R | ND | S | S | S |
| 60 | 22F | S | S | R | ND | S | S | S |
| 49 | 23A | S | S | R | ND | S | S | S |
| 84 | 33A | S | S | R | ND | S | S | S |
| 114 | 35B | S | S | R | ND | S | S | S |
| 29 | 45 | S | S | S | ND | S | R | S |

|  | MDR (resistant to 3 or more classes) |
| --- | --- |
|  | Resistant to 2 classes |

S = Sensitive; R = Resistant; ND = Not done
